# Consuming cholera toxin counteracts age-associated obesity

**DOI:** 10.18632/oncotarget.27137

**Published:** 2019-09-17

**Authors:** Bernard J. Varian, Theofilos Poutahidis, Gordon Haner, Alex Hardas, Vanessa Lau, Susan E. Erdman

**Affiliations:** ^1^Division of Comparative Medicine, Massachusetts Institute of Technology, Cambridge, MA 02139, United States; ^2^Department of infectious Diseases and Pathology, Faculty of Veterinary Medicine, Aristotle University of Thessaloniki, 54124, Greece

**Keywords:** body weight, mouse, exotoxin subunit B, CLS, inflammation

## Abstract

During the past forty years there has been an inexplicable increase in chronic inflammatory disorders, including obesity. One theory, the ‘hygiene hypothesis’, involves dysregulated immunity arising after too few beneficial early life microbe exposures. Indeed, earlier studies have shown that gut microbe-immune interactions contribute to smoldering inflammation, adiposity, and weight gain. Here we tested a safe and well-established microbe-based immune adjuvant to restore immune homeostasis and counteract inflammation-associated obesity in animal models. We found that consuming *Vibrio cholerae* exotoxin subunit B (ctB) was sufficient to inhibit age-associated obesogenic outcomes in wild type mice, including reduced crown-like structures (CLS) and granulomatous necrosis histopathology in fat depots. Administration of cholera toxin reduced weight gain irrespective of age during administration; however, exposure during youth imparted greater slenderizing effects when compared with animals receiving ctB for the first time during adulthood. Beneficial effects were transplantable to other obesity-prone animals using immune cells alone, demonstrating an immune-mediated mechanism. Taken together, we concluded that oral vaccination with cholera toxin B helps stimulate health-protective immune responses that counteract age-associated obesity.

## INTRODUCTION

The global burden of chronic inflammatory diseases is increasing at alarming rates [1, 2]. The continuous rise of obesity, cardiovascular and chronic respiratory diseases, diabetes, infertility, allergy and autoimmunity, cancer, and central nervous system (CNS) dysfunctions, including anxiety and autism, appears to link with modernized lifestyle but remains inexplicable [3–5]. Accumulating evidence suggests that their pathogenesis consistently links with chronic inflammation [6,7]. Underlying systemic immune imbalances linked with bacteria residing in the gut have been proposed as a probable cause of obesity [8,9].

In this context, obesity is one of many chronic inflammatory diseases associated with modernized living. Important effects of gut microbiota in mammalian physiology, including metabolism and CNS functions, place gut microbe-immune cell interactions in the hypothetical center of chronic inflammatory disorders such as obesity [7, 8] [10–18]. An intriguing microbe-centric theory builds upon the so-called “hygiene hypothesis” and proposes that modernized domestication practices including sterile births, refined diets, and antibiotics, result in too few microbe exposures that reduce microbial diversity and favor gut bacterial dysbiosis, ultimately leading to a wide array of systemic health disorders.

Among these, clinical and experimental data suggest that white adipose tissue of obese animals is persistently inflamed [19, 20]. Inflammatory cells are recruited to fat depots and surround dead adipocytes, thus creating crown-like structures (CLS). These are thought to be key initiators in the pro-inflammatory cascade encountered in obesity [19, 21, 22]. At the same time, anti-inflammatory elements of the immune system, such as regulatory T cells, down-regulate host inflammatory responses and normally serve to counteract fat accumulations [19, 20, 23–27]. It follows logically that factors stabilizing immunity in a balanced way will inhibit inflammation-associated adiposity.

In this regard, postbiotic gut bacterial fractions used for oral immunizations have been found to stabilize the immune system and counteract destructive inflammatory responses later in life in both humans and animals. A notable anti-inflammatory paradigm is that of *Vibrio cholerae* exotoxin [28–30]. Immune adjuvant properties of cholera-toxin (ct), make it an attractive tool for induction of tolerance that stabilizes the immune system [31, 32]. The powerful adjuvant and oral tolerance- inducing properties of ct and it’s B subunit (ctB), which is biologically safe and is widely used in neurobiology [30–35], have been shown to prevent experimentally induced inflammatory diseases in various preclinical rodent models [32, 36–38]. Once commonplace worldwide, *V. cholerae* is also a good target for investigating “hygiene hypothesis”-associated phenomena in human subjects, and ctB may be introduced orally in safe, non-pathogenic doses to induce immune stability [31, 32].

Here we used wild type mouse models predisposed to age-associated weight gain, and found that cholera toxin subunit B administration alone was sufficient to prevent development of obesity by an immune-mediated mechanism. An oral vaccination strategy to counteract obesity is practical and safe, with potential of major impact for public health initiatives.

## RESULTS

### Feeding cholera toxin B [ctB) is sufficient to inhibit age-associated obesity in mice

Systemic immune imbalances arising from the gut have been proposed as a probable cause of obesity [8]. We reasoned that microbe post-biotics that stabilize gut immunity will inhibit adiposity. Along these lines, cholera-toxin subunit B (ctB), a major immune stimulatory factor of *Vibrio cholerae*, has powerful immune adjuvant properties when delivered orally, making it an attractive tool for this purpose [30–35]. Thus, we tested whether introducing low doses of ctB will serve to block adipogenesis later in life.

**Figure 1:**
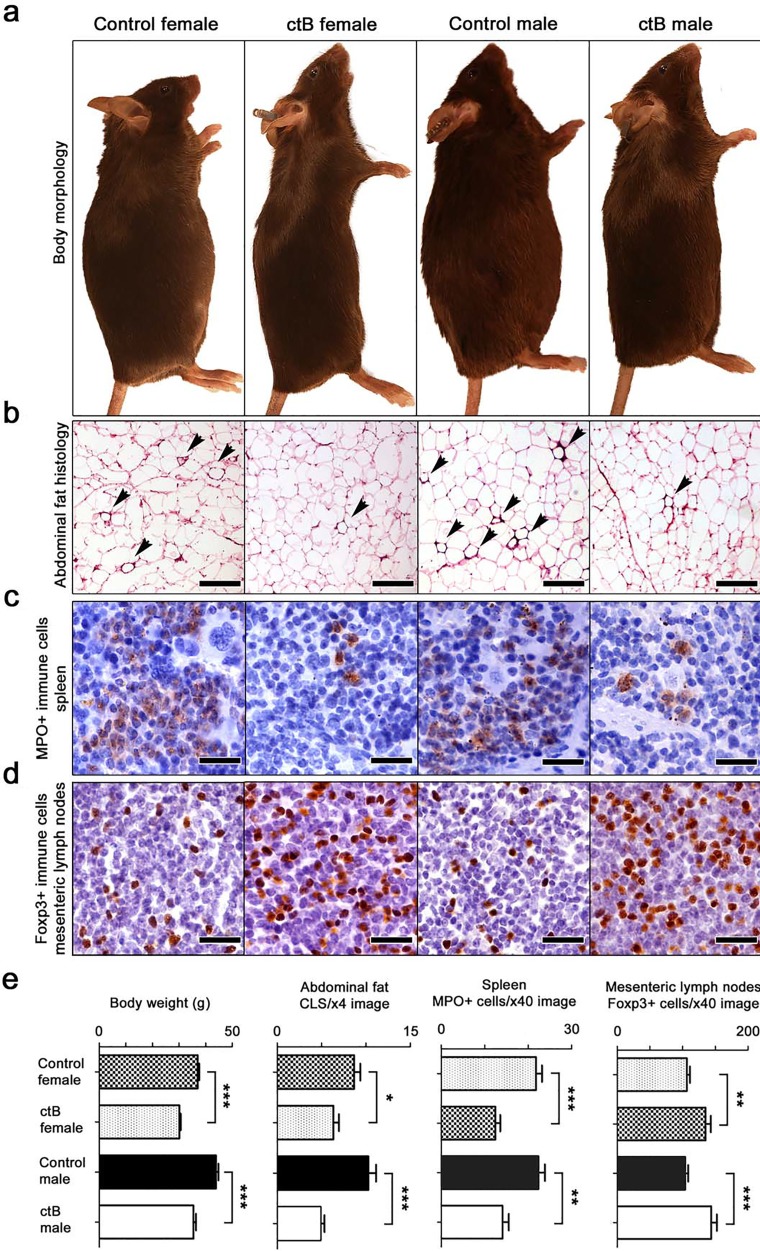
Early-life intervention with ctB rescues mice from age-associated obesity and inflammation. Shown are data collected from C57BL/6 mice of both genders at the age of 9 months. **(a)** At gross examination of whole body morphology, both female and male mice that consumed low doses of ctB eight months earlier have leaner physiques by comparison with untreated controls. **(b)** Treated mice also have less crown-like structures (CLS, arrow-heads), caused by adipocyte death-related inflammation, in their abdominal fat; **(c)** less myeloperoxidase-positive (MPO) granulocytes in their spleens; and **(d)** more anti-inflammatory Foxp3-positive regulatory T cells in their mesenteric lymph nodes compared to control mice. **(e)** Body weight and histomorphometrical analyses shows that the long-lasting effects of ctB are statistically significant. (a) Hematoxylin and Eosin. Scale bars: 250 μm. IHC; Diaminobenzidine chromogen, Hematoxylin counterstain. Scale bars: 25 μm. (b) Numbers on the y-axis of bar graphs correspond to the mean±SEM of the parameter assessed; ^*^p<0.05, ^**^p<0.01, ^***^p <0.001.

First, using C57BL/6 wild type mice, we discovered that three doses of ctB every-other-week at 10 ug per mouse starting at four-weeks-of-age prevented age-associated body weight gain when examined upon necropsy at nine months of age. Specifically, using N=8 mice per group, we found differences in body weights in control male mice = 35.38±2.93g versus ctB-treated male mice = 43.86±2.94g (p<0.001), and control female mice=36.99±0.58g versus ctB-treated female mice=30.09±0.52g, (p<0.001) (Figures 1a and 1e). This indicated significantly less weight gain after consuming ctB during youth in mice offered an *ad libitum* control chow diet.

### Slenderizing effects of ctB were generalizable to other mouse genetic backgrounds

**Figure 2:**
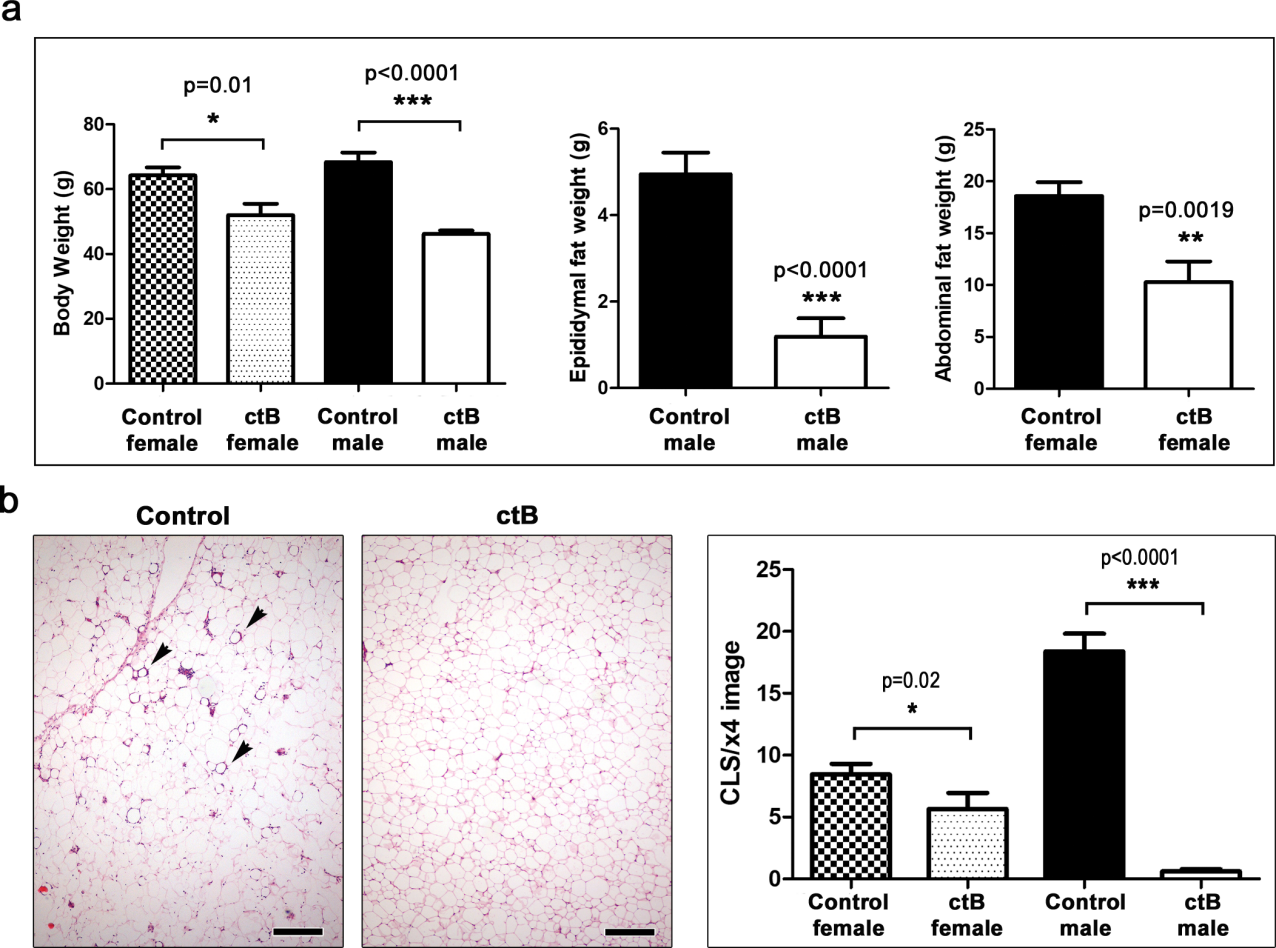
Early-life oral dosing with ctB protects CD1 outbred mice from obesity later in life. **(a)** At the age of nine months ctB-treated mice of both genders have significantly lower body and abdominal fat deposit weights and **(b)** less CLS lesions (arrows) in their abdominal fat compared to untreated controls. The effects are more pronounced in males.(a and b) Numbers on the y-axis of bar graphs correspond to the mean±SEM of the parameter assessed; ^*^p<0.05, ^**^p<0.01, ^***^p <0.001. (b) Hematoxylin and Eosin. Scale bars: 250 μm.

In order to determine if ctB effects were specific to C57BL/6 strain mice, we next examined feeding with ctB in outbred CD1 stock mice. The same ctB dosing regimen as above was applied to eight male and eight female CD1 mice, originally from Charles River Labs, starting at 4–6 weeks of age. As before, at the age of nine months we found differences between control male mice = 68.4±2.9g versus ctB-treated male mice = 46.15±1.1g (p<0.001), and control female mice=64.22±2.5g versus ctB-treated female mice=51.99±3.51g, (p<0.05). In addition, abdominal (epididymal) fat pads in male ctB-treated mice=4.94±0.5g were significantly (p<0.001) smaller after ctB treatment (1.18±0.42g). Likewise, in female mice the abdominal fat in control mice was 18.6±1.33, whereas in ctB-treated animals was 10.27±2g (p<0.01) (Figure 2a). This showed that mice were not only more slender, but also had less abdominal fat accumulation after ctB therapy earlier in life. Thus, ctB outcomes of significantly leaner animals were generalizable to both sexes and diverse genetic backgrounds.

### Cholera toxin B therapy lessened abdominal fat pathology

It was previously shown that gut-driven immune imbalances manifest as extensive fat histopathology during obesity [8]. Here we found that abdominal fat examined histologically at nine months of age revealed increased crown-like structures (CLS) in both C57BL/6 (Figures 1b and 1e) and CD1 mice (Figure 2b), including a type of pyogranulomatous inflammation characteristic of obesity in humans (Supplementary Figure 1) [19]. Although both genders exhibited significant decreases in fat pathology after receiving ctB, differences in both CLS and granulomatous necrosis inflammatory lesions (Supplementary Figure 1) were more pronounced in male mice than in female mice when examined at nine months of age (Figures 1b, 1e and 2b).

### Dosing with ctB restructures host systemic immunity

**Figure 3:**
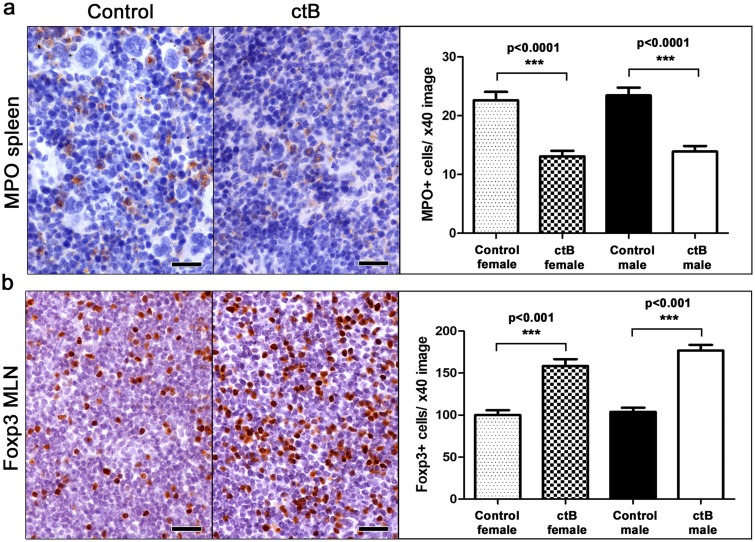
Early-life oral dosing with ctB has lifelong effects in immune cell population host profile. At the age of nine months, both female and male ctB-treated CD1 mice have significantly less **(a)** myeloperoxidase-positive (MPO) granulocytes in their spleens and **(b)** Foxp3-positive regulatory cells in their mesenteric lymph nodes. IHC; Diaminobenzidine chromogen, Hematoxylin counterstain. Scale bars: 25 μm. Numbers on the y-axis of bar graphs correspond to the mean±SEM of the parameter assessed; ^***^p <0.001.

Recognizing that abdominal fat pathology was previously linked with dysregulated immunity, we examined indicators of systemic inflammation in the spleen and mesenteric lymph nodes (MLN) of inbred C57BL/6 and outbred CD1 male and female mice in various treatment groups. We found significantly more myeloperoxidase (MPO)-positive cells in the spleen of control compared to ctB-treated mice in both C57BL/6 (Figure 1c and 1e) and CD1 mice (Figure 3a).

Based on earlier findings that lymph nodes draining the gut display evidence of enhanced immune tolerance after ctB treatment [8, 39], we next examined mesenteric lymph nodes (MLN) of ctB-treated and sham-dosed mice. As predicted, there were significantly more Foxp3+ regulatory T (Treg) cells present per high power microscope field in mice after treatment with ctB (Figures 1d, 1e and 3b).

**Figure 4:**
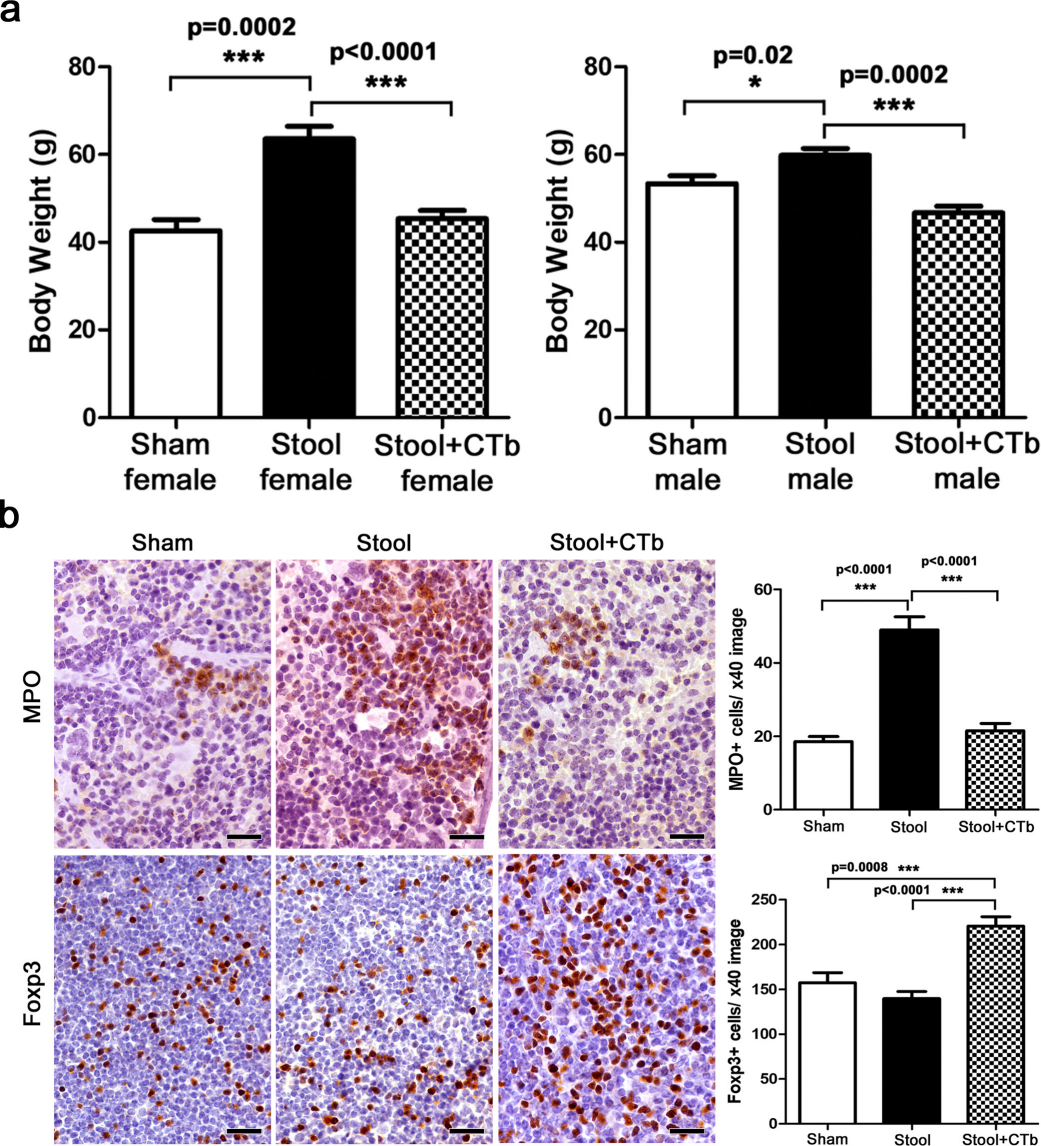
ctB during youth counteracts microbiota-induced obesity. **(a)** After oral consumption of obese mouse stool flora at early life, female and male CD1 Elite mice present with significantly increased body weights at 6 months of age compared to sham-treated controls. This effect is negated by early-life treatment with ctB **(b)** In the same mice, in addition to body weight, ctB also keeps in-check obesity-associated immune cell imbalances, such as MPO-positive granulocyte increase and Foxp3-positive regulatory T cell decrease in lymphoid organs. (a and b) Numbers on the y-axis of bar graphs correspond to the mean±SEM of the parameter assessed; ^*^p<0.05, ^***^p <0.001. (b). IHC; Diaminobenzidine chromogen, Hematoxylin counterstain. Scale bars: 25 μm.

To further test this concept of immune tolerance to microbiota, we performed transfer of gut microbiota in stool samples from obesity-prone CD1 mice. Recipient mice were CD1-Elite (Charles River, Wilmington MA) otherwise harboring a restricted flora microbiome. As predicted based on earlier studies [40, 41], this revealed that the obese-mouse microbiome was sufficient to trigger obesity and inflammation at the age of six months (Figure 4a) by comparison with sham-dosed control mice. At four-weeks-of-age, half of the obese-microbiome recipient mice were randomly subdivided and dosed with ctB while the other half got only saline gavage. When examined at six months of age, we found only animals receiving ctB during youth resisted obesogenic microbiota-induced age-associated weight gain (p<0.001) (Figures 4a, 4b).

Interestingly, feeding with ctB robustly lowered neutrophil counts to baseline levels in more slender mice (*P* <0.001) (Figure 4b). Similarly, ctB administration consistently generated increases of Foxp3+ Treg cells in MLN (*P* <0.001) (Figure 4b). Taken together, these data match earlier findings of Feuerer et al (2009) who observed that protective Foxp3+ Treg cells are associated inversely with adiposity [42].

### Cholera toxin-triggered protection from fat pathology is transferable to naïve hosts via immune cells

**Figure 5:**
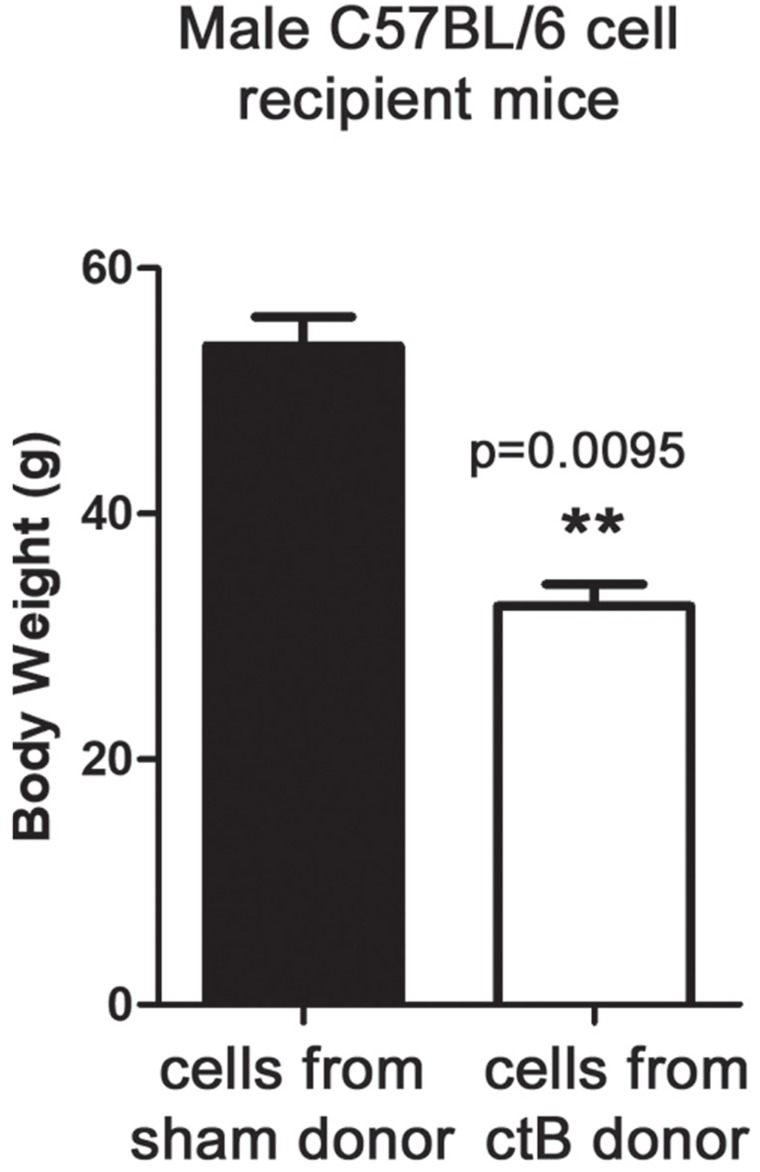
ctB control of age-associated increase of body weight is mediated by immune cells. At the age of 9 months, C57BL/6 male mice that were adoptively transferred at the age of 12 weeks with mesenteric lymph node cell populations from ctB-treated donors, showed statistically significant benefits in age-associated body weight gain control compared to recipients of non-ctB exposed MLN cells. Numbers on the y-axis of bar graphs correspond to the mean±SEM of the parameter assessed; ^*^p<0.05, ^**^p<0.01, ^***^p <0.001.

Based upon our earlier work [8, 39] and that of others [42] showing pivotal roles for immunity, we postulated that ctB protect from obesity by induction of immune tolerance and maintenance of homeostasis. To test whether immune cell balance was sufficient for reduced fat accumulation we used adoptive transfer of mesenteric lymph node cells from male ctB-treated or sham-dosed animals into male naïve syngeneic C57BL/6 mice. For these experiments, cell donors were fed ctB by gastric gavage. We found that recipients of immune cells from donor mice getting ctB treatment had reduced body weights when compared with recipients of cells from untreated control mice (Figure 5). These data showed that the ctB-imbued protection from obesity resided in lymphoid tissue associated with gut interactions and was transplantable to other animals, highlighting a tractable immune-mediated strategy with potential for lowering risk of obesity.

### Oral consumption of ctB later in life also protects against obesity

**Figure 6:**
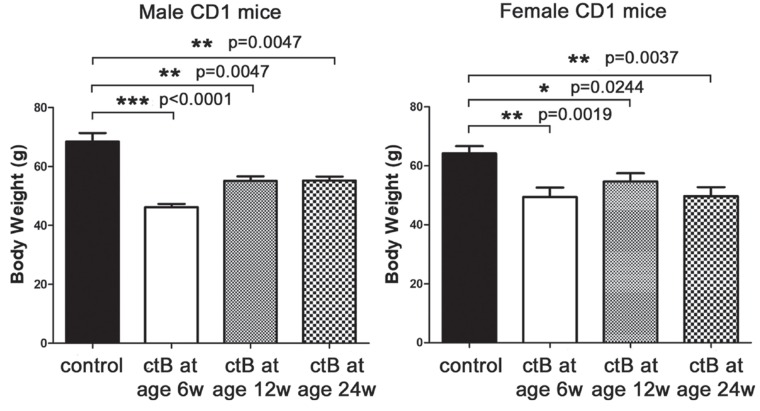
Beneficial effects of ctB do not strictly depend on its early-life consumption. Male and female CD1 mice that were treated with ctB at their early life (six weeks), have significantly different body weights at nine months of age compared to untreated controls. Although to a lesser degree, especially in males, the same beneficial effect is also observed in mice treated with ctB as late as at the age of 12 or 24 weeks. Numbers on the y-axis of bar graphs correspond to the mean±SEM of the parameter assessed; ^*^p<0.05, ^**^p<0.01, ^***^p <0.001.

Finally, to test whether aged adult mice would similarly benefit from dosing with ctB for the first time during adulthood, we used the CD1 mouse model and treated eight mice per group with ctB at 12- or 24-weeks-of-age to compare with male and female mice treated with ctB at youth (Figures 1, 2 and 4). Using the same study design as above, body weights were significantly reduced in all groups of mice treated with ctB. However, male mice were leaner when dosed at a younger age within the weeks after weaning (Figure 6). Thus, there was a benefit to exposures to ctB earlier in life; however, this early life benefit was significant onlyi n male animals (Figure 6). This raises the possibility of using ctB therapy not only during childhood but also during adulthood to lower risk for developing obesity.

## DISCUSSION

We used animal models to test our hypotheses involving immune adjuvant cholera toxin on development of obesity. Mice consuming *ad libitum* standard rodent diets mimicked age-associated adiposity patterns previously linked with gut microbiota in animal models and in people [43]. In the present study, wild type mice with different genetics were fed purified cholera exotoxin B subunit (ctB) - a mucosal immunomodulatory component of Dukoral® vaccine used in humans for cholera diarrhea prevention [44]. Importantly, we show that slim physique outcomes were achievable in both male and female animals of differing genetic backgrounds, indicating potentially broad translational relevancy for human subjects. Lean phenotypes were transplantable to naïve syngeneic animals when using immune cells gathered from mesenteric lymph nodes (MLN) of ctB-treated mice. These observations led us to propose a mechanistic model whereby ctB interacts with the local gut immune system and resident microbiota to stimulate local immune tolerance [45, 46], which in turn imparts systemic immune homeostasis and lowers risk for obesity [8, 42]. Once initiated early in life, gut homeostasis and resiliency to environmental challenges may then become self-sustaining.

We demonstrate that consumption of ctB has a potent slenderizing effect in wild type animals with a competent immune system. In earlier studies, Poutahidis et al (2013) showed that microbial reprogramming of the immune system was pivotal in suppressing systemic inflammation and lowering risk for obesity [8]. According to their hypothesis, probiotic gut microbiota stimulate a vibrant immune response that ultimately recruits regulatory T cells (Treg) that restrain inflammation [45–49]. One specific aspect of this intriguing paradigm is a requirement for balanced and reciprocal activities of pro-inflammatory and anti-inflammatory cells and factors [31, 32]. Interestingly, inter-related roles may exist for Treg cells and thyroid function in obesity [50]. Further data supporting a role for Foxp3+ Treg agree with Feuerer et al (2009), who discovered anti-inflammatory Treg arise inversely with adiposity and insulin resistance in mouse models [42]. Along mucosal surfaces, Treg imbue tolerance that restrains inflammation and imparts local and systemic homeostasis [51]. Along these same lines, asthma and autoimmune diseases have been linked with compulsive Westernized hygiene with insufficient microbe-immune stimulation to induce effective counter-regulatory Treg [52, 53]. In the present paradigm, *Vibrio cholera,* once commonplace throughout the world, is a good choice to restore immune stimulation otherwise lacking in Westernized living. In the situation of ctB, peripheral mucosal immune tolerance, mediated by inducible Treg and tolerogenic antigen-presenting cells, is thought to be responsible for this phenomenon [32], [54, 55]. Using a sterile subunit of *V. cholerae* to jumpstart immunity and induce tolerance [31, 32] is a safe and tractable alternative to viable bacteria. In this way, cholera toxin subunit B has been convincingly shown to protect against diverse inflammatory disorders in animal models and in humans [30, 32–35, 56], giving this broad applicability for public health goals.

One mechanism of tolerance involves oral-pharyngeal exposures to adjuvants. In the present study, we introduced ctB via gastric gavage, interrupting some aspects of oral tolerance in our murine models. In humans, ctB is administered by mouth in Dukoral® vaccine to protect against cholera. Future studies will examine in mouse models whether adding ctB to mouse drinking water enhances protection against obesity. It is unknown whether Dukoral® vaccine provides better protection from obesity than does ctb alone.

Such preclinical studies examining protective roles of ct should not be restricted to age-associated obesity, as in our present study, but also involve diet-induced obesity as well. Mice fed with a high level of fats and carbohydrates have been widely used and provided important clues in the interrelated pathology of obesity, inflammation and metabolic diseases [6, 19, 20]. Interestingly, diet- and senescence-induced obesity share many aspects of pathogenesis, which are collectively presented in two recent detailed reviews [57, 58]. One important commonality is that energy-rich diets cause a subclinical, chronic, low-grade systemic inflammatory state, which is similar to what is caused by immunosenescence in both aged animals and humans [57, 58]. In our previous line of research using probiotic bacteria to counteract the deleterious effects of a Western-style obesogenic diet in mice, we observed mice consuming the high-fat diet had a dramatic decrease in the populations of Tregs in mesenteric [8] and mammary lymph nodes [59]. This dietary-induced effect on Treg is consistent with what has been previously reported [27] and comparable with the age-associated decline in lymph node Treg observed in the present study, where mice consume a normal diet. Overall, the effect of ct on expanding anti-inflammatory Tregs is reported here and in previous studies [31, 32, 39, 55]. Powerful systemic immunomodulatory properties involving various immune cell populations including antigen presenting cells and lymphocytes are well substantiated [28–35, 54]. Taken together, rodent models of diet-induced obesity are also a suitable experimental platform for testing the efficacy of oral ct in ameliorating immunometabolic disorders.

Immunological tolerance involves muted responses of the immune system to self or environmental antigens, including gut bacteria. This process limits collateral tissue damage and is important for physiological well-being. The concepts of homeostasis and immune tolerance are indeed an oversimplification of a complex multi-faceted immune recruitment mediated by ctB. An interesting paradox exists in that stimulating beneficial inflammation is essential for healthful responses to environmental challenges; however, uncontrolled inflammation increases risk of many diseases [60–62]. Under conditions of homeostasis, feedback loops involving Treg apparently permit beneficial immunity yet minimize collateral damage and rapidly restore balance afterwards [11, 63]. Thus, ctB efficiently balances the pro- and anti-inflammatory aspects of the immune system.

Along the same lines, our previous studies show benefits of cholera toxin in inflammation-promoted mouse colon carcinogenesis [39]. In those studies, we found that the toxin was linked most significantly with neutrophils and Tregs. Specifically, a significant increase of Tregs in both colonic mucosa and MLN, co-exist with decrease of colonic mucosa neutrophils was found to persist for several months after cholera toxin consumption [39]. A similar long-term effect on the same immune cells is linked with the favorable outcome of ctB administration observed in the present study. A similar reciprocal immune paradigm also coincided with health promoting effects of probiotic and postbiotic microbe therapies [8, 14, 50, 64–66].

High numbers of neutrophils and their increased activation has been previously reported in young male African American individuals predisposed to obesity [48]. A recent study also highlighted the role of neutrophils in childhood obesity. Specifically, by comparison with normal-weight children, obese subjects had larger numbers of circulating neutrophils with unbalanced Tgf-β and IL-10 expression profiles [47]. Taken together this evidence suggests that gut bacteria or their sterile immunogenic antigens (ie., ctB) may help break the vicious circle of low grade inflammation and obesity [8]. This beneficial effect may be, at least in part, linked with the downregulation of systemic neutrophilic responses.

Another immune cell population of T-lymphocytes, specifically Th17 cells, have also been reported to counteract obesity in mice; however, Th17 cells are proposed to act by shaping a gut microbiota that promotes leanness [57]. Future studies involving gut microbiome analysis may determine in more detail whether consumption of ctB directly affects gut microbial communities. One recent study suggests that this interaction may even involve direct effects of ctB on gut bacteria. Indeed, Patry et al. show that ctB) inhibits *Campylobacter jejuni* growth in the intestine of poultry by blocking its GM1 mimickry and compromising the permeability of its cell membrane [53].

Although we discovered dramatic benefit after early-life exposures to ctB, mice were also significantly slimmer when dosed with ctB for the first time during adulthood at 12-wks-of-age or 24-wks-of-age. This raises the possibility of substantial weight loss benefit after consuming ctB for the first time even during adulthood. It remains to be tested whether initial dosing during infancy with adjuvant boosters later in life will further reduce body fat and optimize weight management potential.

In summary, feeding of ctB alone or in combination with unfamiliar microbiota offers potentially potent, cost-effective and practical options for public weight management. This type of microbe-immune re-programming may ultimately target other diseases linked with obesity and inflammation such as diabetes [19], multiple sclerosis [64], and cancer [25]. Indeed, systemic immune imbalances related to failure of tolerance have been proposed as a cause of extra-intestinal cancer linked with bacteria residing in the gut [11]. Using a mouse model of colon cancer, we have earlier shown that the oral administration of cholera toxin at the early stages blocks colonic polypoid adenoma formation [39, 67]. Other studies have found similar outcomes in animals [68] and in humans [69]. It remains to be seen whether this gut immune-centric strategy broadly translates to successes in the clinic; however, the versatility of ctB to manipulate immune responses make this protein a promising adjuvant for vaccine development to combat a growing Westernized public health crisis.

## EXPERIMENTAL METHODS

### Animals

Genetically inbred *wild type* C57BL/6 strain mice (Jackson Labs, Bar Harbor, ME) and outbred CD-1 mice (Charles River; Wilmington, MA), were housed and handled in Association for Assessment and Accreditation of Laboratory Animal Care (AAALAC)-accredited facilities with experimental methods and housing as specifically approved by the Institutional Animal Care and Use Committee. Mice were bred in-house to achieve experimental groups. The basic experimental design was to expose eight male and eight female mice to cholera toxin subunit B (ctB) starting at four-weeks of-age. Other groups of mice were first dosed with ctB at age = 12 weeks or age = 24 weeks, and in all cases monitored until euthanasia using carbon dioxide overdose at nine months of age, unless otherwise specified. Each experiment included 5-15 animals per group with one replicate experiment (total N=10-30 mice per group). Obesogenic microbiome exposure used fresh stools in CD1-Elite (Charles River; Wilmington, MA) mice at 3-4 weeks of age, with terminal body weights measured at 6 months of age. Studies involving adoptive immune cell transfers into syngeneic mice occurred at 3 months of age. Tissues were collected upon necropsy.

### Diets

Mice in all experiments were fed *ad libitum* a diet of standard mouse chow ProLab IsoPro RMH 3000 (Lab Diet; St Louis MO).

### Dosing with cholera toxin B

To test whether dosing with cholera toxin B (ctB) was sufficient to inhibit age-associated obesity, mice were dosed 0.2ml by gastric gavage three times every-other-week with 10 μg of ctB. For this purpose, lyophylized ctB (MilliporeSigma; Burlington MA) was reconstituted with sterile PBS to achieve a final dosing volume of 0.2ml per mouse.

### Adoptive transfer of immune cells into recipient mice

Knowing that GI tract origin inflammatory response is associated with obesity in our model, we hypothesized that cells within gut-associated lymph nodes (MLN) may be pivotal in etiopathogenesis. In order to test this hypothesis, we utilized cell transfer of MLN collected from *ctB-dosed* or sham-dosed donor *wt* mice (Figure 5). For this experiment, *C57BL/6* mice of age 12 weeks underwent ip injection of 5X10^6^ single cell suspension in HBSS per mouse of cells gathered from mesenteric lymph nodes of *ctB* or sham saline-dosed *C57BL/6 wt* mice, as described above. Adoptive transfer experiments were conducted using two separate trials with 6 mice per trial.

### Histopathology and immunohistochemistry

For histologic evaluation, formalin-fixed tissues were embedded in paraffin, cut at 5 μm, and stained with hematoxylin and eosin (HE) or immunohistochemistry (IHC). For MPO- and Foxp3-specific IHC, rabbit polyclonal antibodies against myeloperoxidase (ThermoFisher Scientific/Lab Vision, Fremont, CA) and rat monoclonal antibodies against Foxp3 (eBioscience, Inc., San Diego, CA) were used as primary antibodies. Heat-induced antigen retrieval was performed with citrate buffer, pH 6, for myeloperoxidase (MPO) or with EDTA buffer, pH 8, for Foxp-3. Rabbit primary antibody binding was detected with goat anti-rabbit polymer HRP (ZytoChem Plus, Berlin, Germany) whereas rat antibody binding was detected with species-appropriate biotinylated secondary antibodies (Serotec, Oxford, UK). Color was developed with Untravision DAB substrate-chromogen system (ThermoFisher Scientific/Lab Vision) and tissues were counterstained with hematoxylin.

Lesions and IHC-labeled immune cells were identified and quantified by a pathologist blinded to sample identity. Histomorphometrical analysis was done in tissues of eight randomly selected mice per experimental group. Crown-like structures (CLS) in HE-stained abdominal fat sections or immunopositive cells in IHC-stained mesenteric lymph node were assessed as previously described (8). Briefly, multiple images of comparable histological fields were taken at x4 magnification for CLS or at x40 high magnification for immune cell counts. Three images were randomly selected form each mouse resulting in eighteen images from each experimental group for counting. Results were recorded as numbers of CLS or IHC-positive cells per image. For all morphometrical counts the Image J image processing and analysis program (NIH, Bethesda, MD) was used.

### Statistical analyses

The Mann-Whitney U test was used for body weight, diet, calorie consumption, and histomorphometry. A p-value < 0.05 was statistically significant.

## SUPPLEMENTARY FIGURE


