# Supplementary material for: Consuming cholera toxin counteracts age-associated obesity
Source: Oncotarget. 2019 Sep 17;10(53):5497–509. doi: 10.18632/oncotarget.27137 (PMC6756858; doi:10.18632/oncotarget.27137)
Supplement: Supplementary file 1 [file oncotarget-10-5497-s001.pdf]

Consuming cholera toxin counteracts age-associated obesity

SUPPLEMENTARY MATERIALS

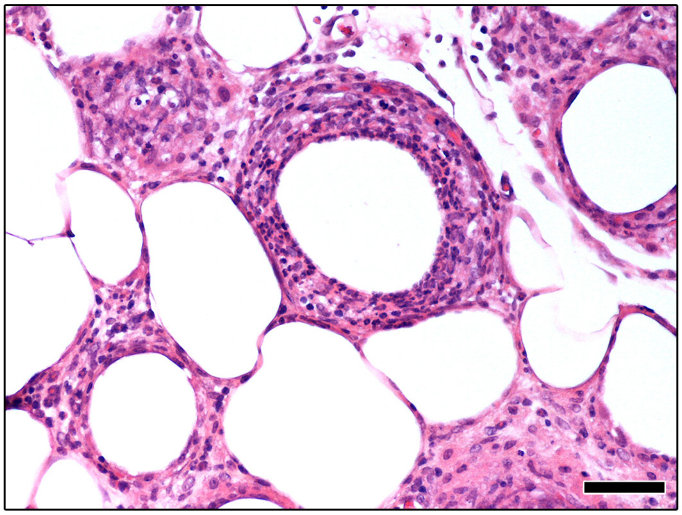

Supplementary Figure 1: Nine-months-old CD1 male mouse. Characteristic focal pyogranulomatous inflammation lesion in the epididymal adipose tissue of obese mouse.Hematoxylin and Eosin. Scale bars: 25 µm.
